# The Body Knows What It Should Do: Automatic Motor Compensation for Illusory Heaviness Contagion

**DOI:** 10.3389/fpsyg.2012.00244

**Published:** 2012-07-13

**Authors:** Tomohisa Asai, Eriko Sugimori, Yoshihiko Tanno

**Affiliations:** ^1^Department of Cognitive and Behavioral Science, Graduate School of Arts and Sciences, The University of TokyoTokyo, Japan

**Keywords:** body resonance, motor simulation, simulation hypothesis, mirror-neuron system, motor compensation

## Abstract

We can share various feelings with others just through observation, as if it were an automatic resonance. This connective function between the self and others could promote the facilitation of our social communication; however, it is still unclear as to how it works in terms of self-other representation. In this study, we showed participants a picture of a model holding a ball, which was weighted with sand. We instructed participants to move one of their arms to a horizontal position and hold it immobile. Those participants who knew the actual weight of the ball (1 kg) tended to raise this arm above the horizontal, in response to their expectation of the need to resist the weight of the ball. This compensatory reaction to the illusion of heaviness suggests that our bodily resonance could be mandatory and predictive. We discuss this new behavioral phenomenon in terms of motor simulation or the mirror-neuron system.

## Introduction

When we are watching movies or home videos, we can enjoy the experiences of a character as if we are undergoing these experiences ourselves. A clear example of this might be a situation wherein a character is in pain, or, additionally, some people may strain themselves when watching weight lifting. Simulation theory might explain such automatic responses, that is, observing another person may automatically generate anticipation of the same experience in oneself (e.g., Jeannerod and Pacherie, 2004; Thioux and Keysers, 2010). Action and perception might be fundamentally coupled (James, 1890; Watanabe, 2008); therefore, observers may have the capacity to simulate a variety of different information that is available from others: tactile sensation (Keysers et al., 2004), pain (Singer et al., 2004), emotional state (Platek et al., 2005; Palagi et al., 2009; de Greck et al., 2012), and motor performance (Calvo-Merino et al., 2005; Lahav et al., 2007; Aglioti et al., 2008). These social cognitive functions that allow us to understand what others are experiencing are often broadly referred to as empathy (Decety and Ickes, 2009), and might be underpinned by neural mechanisms, such as the mirror-neuron system (MNS; Iacoboni, 2009).

Among these, the domain of perception and action, which does not involve emotional reactions, is referred to as bodily resonance, motor contagion, motor simulation, automatic imitation, or direct matching (Iacoboni et al., 1999; Blakemore and Frith, 2005; Brass and Heyes, 2005; Schutz-Bosbach and Prinz, 2007; Aglioti et al., 2008; Liepelt et al., 2010). Some studies have suggested that this simulation of others’ sensation could be “mental re-enaction,” which implies that we simulate according to our own previous experiences (Heyes et al., 2005; Prinz, 2006), because an observer lacking the specific representation of a given feeling may hardly be capable of directly simulating someone experiencing this feeling (that is, correspondence problem; Brass and Heyes, 2005; Singer, 2006). This may be especially true of skilled and complicated actions, such as dancing or piano playing (Calvo-Merino et al., 2005; Lahav et al., 2007), for which specific training is required (Heyes et al., 2005). It seems as though we have the capacity to simulate the action of others if that action is also included in our own repertoire of actions. However, those who have never experienced weight lifting can also simulate the sensations experienced by others undertaking those activities. Therefore, another possibility may be that the simulation is through “predictive encoding or computational interpretation” (Hurley, 2008), and might not be limited to sensations that have already been experienced (Danziger et al., 2009). The interpretation of the actions of others, which are visually identical, but have different contexts, may affect the reactions of observers (Iacoboni et al., 2005), suggesting that we can simulate the actions of others predictively (Blakemore and Frith, 2005) and even estimate background intentions or goals (Liepelt et al., 2008, 2010) as long as those actions are simple (Flanagan and Johansson, 2003; Fogassi et al., 2005). As is obvious, this idea is not contradictory to mental re-enaction theory, because previous experiences could help this predictive computation, especially with regard to skilled actions. A previous study suggested that pro-basketball players, but not big fans of basketball, could predict the future success or failure of the shots of others (Aglioti et al., 2008). Nevertheless, our first hypothesis is that our motor simulation might be realized by predictive encoding of the sensation of others, according to the interpretation of the situation.

These phenomena, wherein we can simulate the sensations of others automatically using our own body, might sound passive and mandatory; therefore, some studies refer to these kinds of illusions as “contagion,” in which we feel non-existent pain by observing others, for example, when we see or hear something non-existent in perceptual illusions (Singer et al., 2004; Watanabe, 2008; Palagi et al., 2009). However, whether this simulation might really be driven mandatorily remains unclear, although some previous studies have suggested that some types of empathy including motor simulation could be driven automatically (Bien et al., 2009; de Greck et al., 2012). In other words, it is a question of whether we ignore the information available from others and inhibit our simulation. This is also essential in terms of the neural mechanism. It is now well established that a neuronal system, named the MNS, exists in both monkeys and humans. During action observation, the neural structures involved in the execution of the observed actions are recruited in the brain of the observer through the MNS, as if that person is the agent of the action (Rizzolatti and Craighero, 2004). If the motor simulation is based on the MNS, which does not distinguish between external (others) and internal (self) action representation, this process should be mandatory. However, a question that often emerges is that why, if this is so, do we not imitate with others all the time (Brass and Heyes, 2005; Pineda, 2008)? Therefore, the MNS probably possesses an inhibitive component, which keeps us from having resonant reactions for everything we see (Brass and Heyes, 2005), because having an automatic process such as this is not always appropriate for effective social behavior (Lee and Tsai, 2010). Therefore, a second hypothesis is that the observation of others would mandatorily affect our own mental state, but that we would simultaneously compensate automatically for this transmitted sensation.

The present study suggests that our motor simulation would be predictive and mandatory, and we attempt to demonstrate this by administering the new illusory phenomenon: heaviness contagion. We showed participants a picture of another person’s hand holding what appeared to be a lightweight ball. In reality, the ball was weighted with sand (1 kg). Participants were instructed to hold their arms in a horizontal position and to keep them immobile. We focused on the arm movements of the participants when they observed another person’s hand holding a ball. In Experiment 1 (A, B), only the group who knew that the ball was heavy raised their arms above the horizontal in response to their expectation of the need to resist the illusory heaviness, suggesting that the heaviness contagion is predictive and mandatory. In Experiment 2 (A, B), we showed that heaviness contagion is driven by observing others (not objects), and in conditions in which the self (participants) and others are in the same situation (i.e., a similarity effect), suggesting that the heaviness contagion might be a possible expression of motor simulation as well as empathy.

## General Method

### Participants

All the participants were right-handed university students (handedness index >8: H.N. handedness inventory (Hatta and Kawakami, 1995), and none of them attended more than one experiment. They were recruited randomly from an introductory psychology class, and written informed consent was obtained from all participants before the experiments were conducted. All participants reported normal or corrected-to-normal vision, hearing, and somatosensation and no neurological abnormalities.

### Apparatus

The experiments took place in a silent, dim room. In order to display the visual stimuli and conduct the experiment, we used MATLAB (MathWorks, Natick, MA, USA). The visual stimuli were presented on a virtual screen through a head-mounted display (Experiment 1A), white board through a projector (Experiment 1B), or PC display (Experiment 2AB). The hand positions of the participants were recorded during the task by using a wireless mid-space mouse (Experiment 1A), a 3D motion-capture device (Experiment 1B), or a high-speed video camera (Experiment 2AB).

### Stimuli

**Figure 1:**
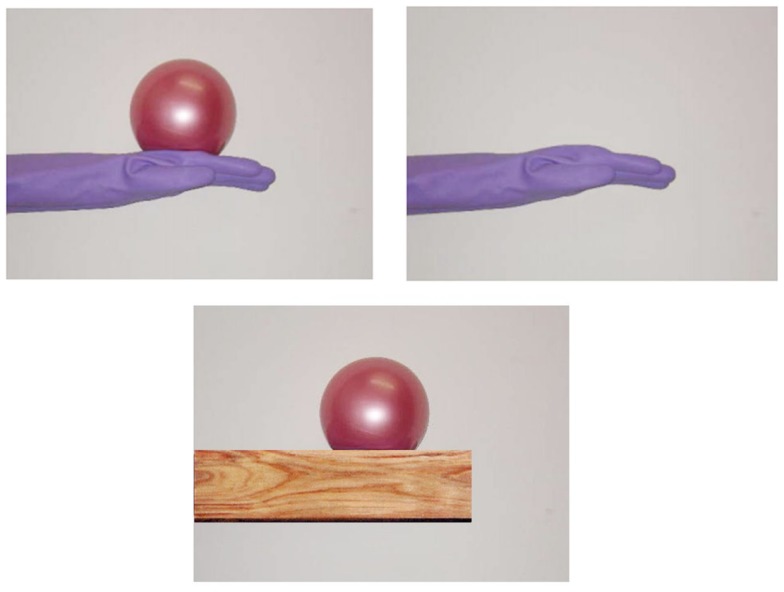
**Visual stimuli used in the present study**.

The visual stimuli consisted of life-sized pictures of a model’s hand holding a ball, as shown in Figure 1. Some previous studies suggest that personal information (e.g., sex, hand size, mole, skin color, etc.) can affect the degree of empathy that participants feel for others (see General Discussion for detail); therefore, in order to exclude such information, the model wore a blue rubber glove. The weighted ball shown in the visual stimuli (Weighted Ball, Regent Far East, Inc., Ashiya, Japan) weighed 1 kg and was 40 cm round. It appeared to be a normal, lightweight rubber ball; however, it was actually filled with sand to add weight. In some conditions, we also used pictures of a hand without the ball, or showed pictures of the ball placed on objects (a wooden block). The weight stimuli were identically weighted balls. Some participants held the ball in their left hand, which was resting on the table, while others held an identical-looking, but light weight (130 g), ball, from which the sand had been removed.

### Procedure

All participants sat in front of the display or screen. Before the experiment began, they received brief training to ensure familiarity with the instruments and experimental requirements. In the experiment itself, we instructed each participant to hold their right hand in a horizontal position throughout the trial, which lasted 30−90 s depending on the experiments. We intentionally manipulated the duration of visual stimuli presentation between experiments in order to suggest duration- or timecourse-independence. The arm was first held out straight, to ensure what was felt to be a horizontal position. When the arm was properly positioned, the visual stimulus appeared. We instructed the participants to remain immobile when the stimulus appeared. We then recorded the height of the hand, if it was raised, throughout the remainder of the trial. After the trial, the participants were asked to lower the hand and relax.

### Data analysis

In order to measure the hand position, we translated the row pixel data into Euclidean distance (i.e., mm), and the starting position was set at zero, so that a positive value of hand height meant that the participant’s hand was raised from its starting position. These values are useful when observing the time course of the hand movement of the participants. Furthermore, we calculated movement velocity (average hand position displacement per second: mm/s) during the task, for comparison among conditions or groups in each experiment. A positive value of movement velocity meant that the position of the hand was being progressively raised during that period.

### Ethics statement

The protocol of the present study was approved by the local ethics committee (The Ethical Committee on Human Experimentation of the Graduate School of Arts and Sciences, The University of Tokyo).

## Experiment 1A

In this experiment, we suggested that automatic predictive compensation would occur in response to a simulated feeling. We hypothesized that participants would raise their hand when observing a person who feels heaviness in the hand because they should predict a compensatory need to adjust to the illusory weight: “heaviness contagion.”

### Material and methods

#### Participants

Forty university students (26 males and 14 females, mean age = 19.0 years, range = 18−21 years) were randomly divided into four groups: the BB (Ball seen, Ball held), BN (Ball seen, No ball held), NB (No ball seen, Ball held), and NN (No ball seen, No ball held) groups. In this experiment, only the weighted ball (1 kg) was used as a prop. For example, those in the BB group saw a model’s hand holding the weighted ball, and held an identically weighted ball in their left hands, whereas those in the NN group saw the model’s hand holding nothing, and held no ball themselves. In the BB and NB groups, participants held the ball and were therefore aware of its weight. In the NN and BN group, participants had no information regarding the weight of the ball.

#### Apparatus

The head-mounted display device (GVD-510-3D, Shenzhen Oriscape Electronic Co., Ltd., Shenzhen, Guangdong, China) was attached to a chin rest, and the participants looked through a device that displayed the image of a 28° visual angle virtual screen. The apparatus was arranged so that it appeared as though the virtual screen was located just beyond the reach of the participants (approximately 60 cm). An eye pad prevented them from seeing their hands, and hand positions were measured every second (1 Hz), using a wireless mid-space mouse (BOMU-W24A/BL, Buffalo, Inc., Nagoya, Japan). This device weighed 135 g and was equipped with a gyroscopic sensor that allowed it to be used in the air.

#### Procedure

Each participant sat in front of the chin rest, on which they each placed their chin. Their right arm was held out straight, using the mouse device to ensure a horizontal position. When the arm was properly positioned, the participant clicked the mouse button once. Following a random interval of 1-2 s, to allow for micro-motions caused by clicking the mouse, the visual stimulus appeared on the virtual screen for 45 s. The task requirement was to remain immobile when the stimulus appeared. Each participant completed a single trial.

#### Questionnaire

After the experiment, participants completed a retrospective questionnaire designed to measure the extent to which they felt as though the hand of the model was their own hand, and therefore actually felt the weight of the ball as presented on the screen. It was explained that the purpose of the questionnaire was to simply provide information regarding impressions of the task, and participants were encouraged to answer freely. It was expected that this instruction would avoid the possibility of an influence of experimenter effects or demand characteristics on responses. The questionnaire consisted of five items, each of which asked for an accuracy rating of a particular statement using a five-point scale. The statements were as follows: (1) It felt as though your hand was weary and numb. (2) It seemed as if the hand on the screen was your own hand. (3) It felt like your hand was moving lower. (4) It seemed as if the ball was in your own hand. (5) Your hand felt the weight of the ball. Participants in NN group had neither seen nor held the ball, so they rated answered for only three statements: Q1, 2, and 3. The topics “resonance with the model’s hand” and “a feeling of weight” were included in questions 2, 4, and 5, and questions 1 and 3 respectively.

#### Results and discussion

**Figure 2:**
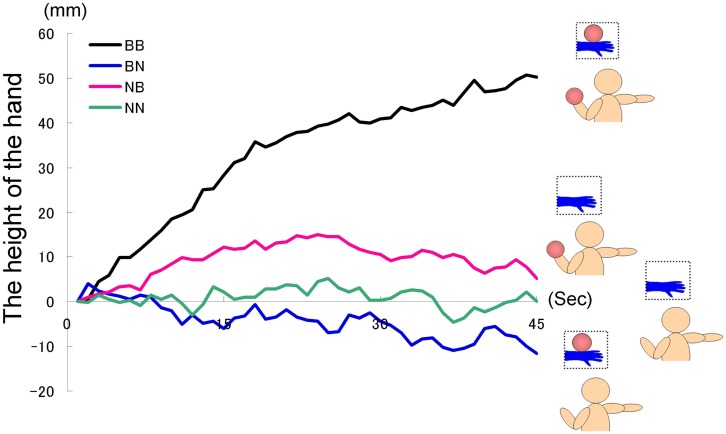
**Time course of the height of the hand in each group in Experiment IA**.

**Figure 3:**
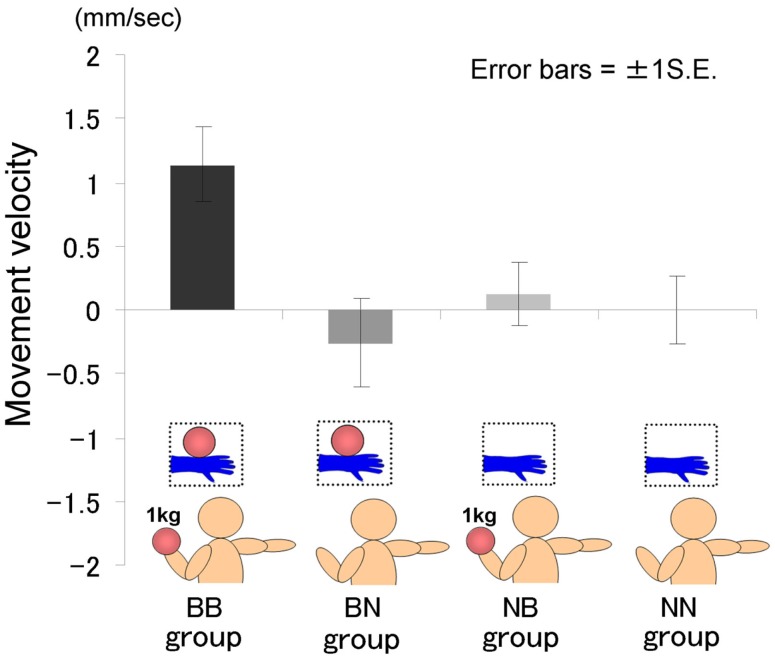
**Movement velocity in each group in Experiment IA**.

The time courses of the hand position of the participants indicated that only those in the BB group tended to raise their right hand gradually, whereas those in the other groups kept their hand almost immobile (Figure 2). A two-way ANOVA (two visual stimuli × two weight stimuli) was conducted to examine the movement velocity of the four groups (Figure 3). These analyses demonstrated a significant interaction [*F*(1.39) = 4.88, *p* < 0.05], significant simple main effect of visual stimuli under the ball-held condition [*F*(1.36) = 6.16, *p* < 0.05], and significant simple main effect of weight stimuli under the ball-seen condition [*F*(1.36) = 11.67, *p* < 0.01]. It is suggested that only those participants who saw a model holding the weighted ball and held an identical weighted ball in their left hands raised their right hand.

**Figure 4:**
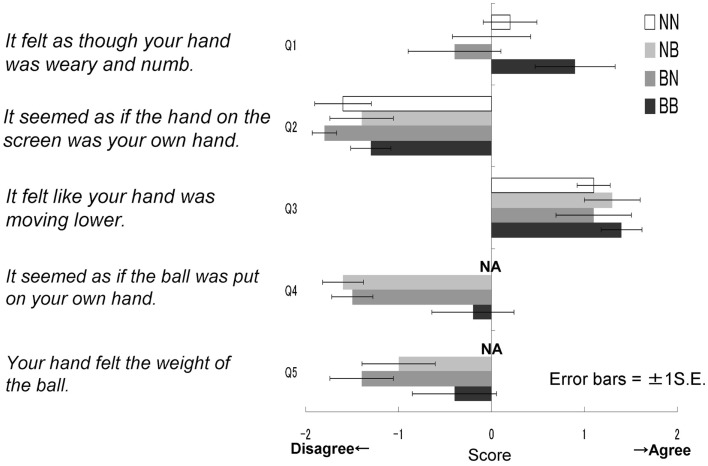
**Questionnaire scores in each group in Experiment IA**. It felt as though your hand was weary and numb. It seemed as if the hand on the screen was your own hand. It felt like your hand was moving lower. It seemed as if the ball was put on your own hand. Your hand felt the weight of the ball.

The results of the questionnaires were then analyzed (Figure 4). The NN group did not answer questions 4 and 5; therefore, for statistical analysis we conducted a two-way ANOVA to all five questions for just three groups (five questions × three groups), omitting the NN group. These results were then analyzed further using Ryan’s multi-comparison method (i.e., R-E-G-W’s *F* test). These calculations revealed significant main effects for groups: *F*(2.27) = 5.99, *p* < 0.01. Main effects for the questions were also significant: *F*(4.108) = 33.73, *p* < 0.01; however, the interaction was not significant: *F*(8.108) = 0.84, *p* > 0.50. Comparisons among the three groups revealed significant differences between the BB and BN groups, and between the BB and NB groups (*p* < 0.01). With regard to the main effect of the questions, Q3 was most often agreed with, followed by Q1; fewer participants agreed with the other three statements (i.e., Q3 > Q1 > Q2 = Q4 = Q5, *p* < 0.05). These findings suggest that the BB group agreed most strongly with the statements related to the feeling of resonance and then heaviness, although in general, the participants did not agree with the statements related to resonance (Q2, 4, 5) compared to those related to the feeling of heaviness (Q1, 3).

The results of hand movement and the questionnaire showed that the participants in the BB group subjectively felt the weight of the ball most heavily. They could have felt a need to adjust to the perceived weight, since they were given instructions to keep their hand horizontal throughout the trial. In the absence of actual weight, we might have expected their hands to move higher as they attempted to compensate for this illusory weight. The finding that participants in the BB group raised their hands over the course of the trial supports the hypothesis that they were compensating for the subjective sense that they were holding a weighted ball. On the contrary, participants in the BN group, who did not know that the ball in the picture was heavy, did not raise their hand. Though we might assume that this is because the BN group predicted that the ball must be as light as it appeared to be, we conducted an additional experiment to address the limitation revealed by this problem.

## Experiment 1B

In this follow-up experiment, minor changes were made in order to examine the dynamic process of heaviness contagion (i.e., a within-participants procedure) as well as entire arm movements (shoulder, elbow, wrist, and fingertip) for a longer period of time (90 s). Furthermore, the no ball group in the previous experiment was replaced with the light-ball group in the present experiment to control for prediction of the weight of a ball in a picture.

### Material and methods

#### Participants

Eight participants (Five males and three females mean age = 27.8 years, range = 22−44 years) were randomly divided into two groups. Both groups saw a model’s hand holding a ball and they also held a visually identical ball in their left hands. We used two balls as the weight stimuli with visually indiscernible differences: one was filled with sand (as in Experiment 1A: 1 kg); the other was not filled with sand (130 g). The first group held a weighted ball (heavy-ball group), whereas the second group held a non-weighted ball (light-ball group). The former group anticipated that the ball in the pictures was heavy, but the latter group anticipated that the ball was light.

#### Apparatus

We refurbished the apparatus, because the previous apparatus appeared to be unique. We used a virtual screen to exclude external noise (i.e., participants could only see the visual stimuli over a black background) in the previous experiment, expecting the participants to feel a sense of immersion. Furthermore, although the mid-space mouse device, which was used to measure hand movement, was not particularly light in weight (135 g), it might nevertheless produce results. In this experiment, the projector device (WT615J, NEC, Tokyo, Japan) presented the visual stimuli on the white board, located 1 m in front of the participants. We measured hand positions using a 3D motion-capture device. Participants attached four infrared reflection markers to the following body parts: shoulder (Position 1), elbow (Position 2), wrist (Position 3), and tip of the middle finger (Position 4). The 3D position of each marker was recorded using a video-based 3D acquisition system, which, in turn, used two high-speed CCD cameras (Himawari CL33; Library, Tokyo, Japan). The sampling rate was 100 Hz; we finally down-sampled to 1 Hz using averaging.

#### Procedure

**Figure 5:**
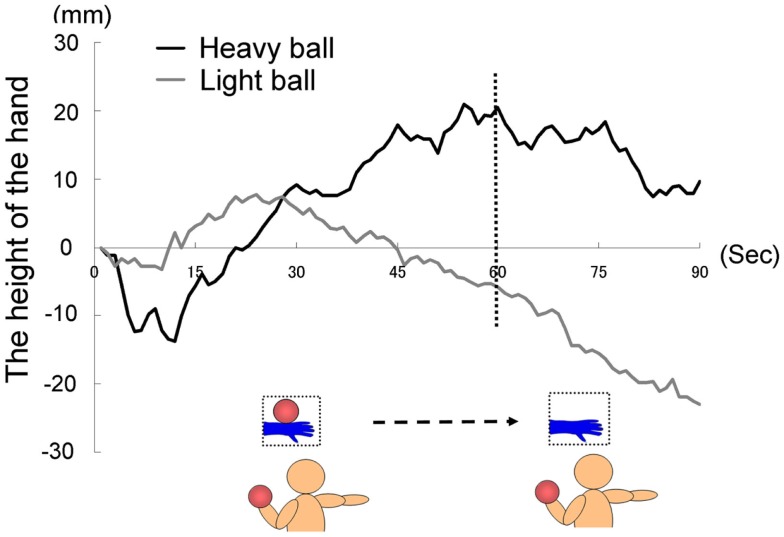
**Time course of the height of the hand (fingertips) in each group in Experiment I B**.

The visual stimuli were presented in front of each participant as they were seated, and they corresponded spatially to each participant’s right arm. In this experiment, the pictures of the hand holding a ball changed mid-course into those of pictures with no ball. As in Experiment 1A, we instructed all the participants to hold their right hand in a horizontal position throughout the trial, which lasted 90 s. Our preliminary experiment suggested that 90 s was the approximate limit that the hand could be held in an approximately horizontal position. Participants were also instructed to look at the visual stimuli, not their hand, as we could not use an occluder, since it could visually block the hand from the video cameras. The right arm was held out straight with fingers stretched in order to ensure a horizontal position during the course of a visual countdown of 3 s. The visual stimulus was presented from the time of zero and the recording of the hand position began. After 60 s, the image of a hand holding a ball was changed to one of a hand with no ball (see Figure 5), that is, a within-participants procedure was used in this experiment, whereas a between-participants procedure was employed in Experiment 1A. The order of the visual stimuli was fixed (that is, “with ball” first, and then “without ball”) in the current experiment because it is possible that the participants would experience muscle fatigue during the latter half of the session (participants who are presented with the “without ball” image first and then the “with ball” image might not raise their hands because of muscle fatigue), which would result in differences between the counterbalanced groups that are not due to experimental manipulation. Each participant completed a single trial where the following body parts were recorded: shoulder, elbow, wrist, and tip of the middle finger.

#### Results and discussion

**Figure A1:**
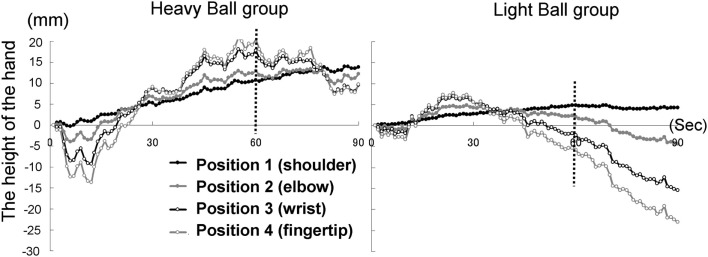
**Time course of the height of the hand (four positions) in each group in Experiment lB**.

The time courses of the hand positions of the participants indicated that the heavy-ball group tended to raise their right hand over their shoulders gradually while observing a model’s hand holding a ball; however, after 60 s, when the image was changed to a picture of a hand without a ball, the hand started to lower. This indicates that the hand raising was based on their shoulder as a fulcrum point, because they might feel heaviness on the back of the hand as if it were the model’s hand. Conversely, participants in the light-ball group lowered their hands gradually (Figure 5; Figure A1 in Appendix).

**Figure 6:**
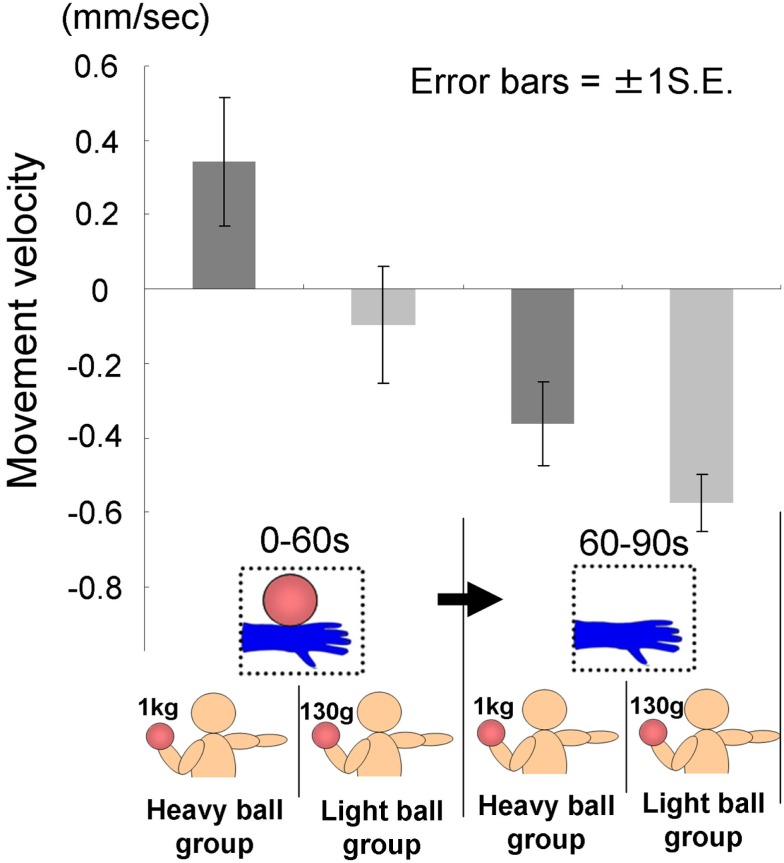
**Movement velocity in each group in Experiment lB**.

We conducted a two-way ANOVA (two groups × two visual stimuli) to examine the movement velocity of the hand (i.e., fingertips; Figure 6). These analyses demonstrated a significant main effect of group [*F*(1.6) = 6.00, *p* < 0.05], and a significant main effect of visual stimuli [*F*(1.6) = 18.49, *p* < 0.01], but non-significant interaction [*F*(1.6) = 0.67, *p* > 0.50]. It is clear that the trend to raise the right hand was observed during the presentation of the image of a model’s hand holding a ball, when participants simultaneously held a visually identical heavy ball in their left hand, suggesting replication of Experiment 1A in a within-participants manner. Conversely, after 60 s, participants in both groups lowered their hands gradually, maybe because of expected muscle fatigue. The present experiment aimed to observe arm movement up to the limit of fatigue; however, there may be confounding between muscle fatigue and hand-lowering, though the rising hands started lowering after just 60 s from the beginning of the experiment (see Figure 5). We addressed this limitation in the following experiments.

Experiment 1B reconfirmed the “heaviness contagion” overall; observation of the model’s hand holding a heavy ball was associated with raising of the hand. This could be driven predictively (merely the prediction of heaviness raises the hand of a participant) and mandatorily (that is why participants must compensate for their illusory heaviness: they did not ignore it). However, a further question must be addressed: which mechanism would cause this phenomenon? The most probable mechanism is direct matching, where we directly map the observed sensation of other agents onto our own sensorimotor representation (Iacoboni et al., 1999). Recent studies have suggested that the direct matching system, which includes motor simulation, bodily resonance, and automatic imitation, might have a biological bias (Press et al., 2005; Tsai and Brass, 2007; Watanabe, 2008; Liepelt and Brass, 2010; Liepelt et al., 2010), indicating that we do not simulate non-human agents. Experiment 2A, with some changes in experimental procedure, was conducted to address this issue. In the current experiment, we presented “with ball” first, followed by “without ball,” and the durations of the two visual stimuli were different (60 s for “with ball” and 30 s for “without ball”) in order to confirm that the raising of the hand would continue for a longer time (as long as “with ball” was presented), compared to Experiment 1A (45 s). In the next experiment, we presented “with ball” in the middle of the session with the same duration as the other visual stimuli conditions.

## Experiment 2A

Our next aim was to show that heaviness contagion could be driven by observing a person, not by observing an object, because we should simulate a co-specific counterpart in terms of MNS. Furthermore, we made some minor changes. A model’s hand without a ball was shown first, followed by the presentation of a model’s hand holding a ball in order to control for hand-lowering caused by muscle fatigue. Participants also repeated trials in this experiment to indicate resistance to habituation.

### Material and methods

#### Participants

A total of 17 participants (6 males and 11 females, mean age = 19.5 years, range = 19−21 years) took part in this experiment; however, one female dropped out because she could not keep her hand in a horizontal position during the trials.

#### Apparatus

The apparatus was changed slightly. In this experiment, we showed the visual stimuli on a 19′′ LCD display (LCD-AD19H, IO-DATA, Tokyo, Japan), located 60 cm in front of the participants. The visual stimuli were presented in front of each participant where they were seated, and corresponded spatially to each participant’s right arm. We measured hand positions using a high-speed camera (EX-FC150, CASIO, Tokyo, Japan), which was located 1 m just behind the right arm when the arm was raised horizontally. The sampling rate was 120 Hz; we finally down-sampled to 1 Hz using averaging. An occluder prevented the participants from seeing their right arm.

#### Procedure

As in Experiment 1, we instructed each participant to hold the right hand in a horizontal position with their fingers stretched throughout the trial, which lasted 60 s. For the first 20 s, the image of a hand without the ball was presented. After 20 s, the image was changed to one of a hand holding a ball. Furthermore, after 40 s, the image of a hand holding a ball was changed to one of a ball on a wooden block. The first and second images were the same as those used in previous experiments, whereas the third was newly prepared, so that the size of wooden block was approximately the same as a model’s hand. All participants held a weighted ball (1 kg) in the left hand during each trial to ensure that they were aware of the weight of the ball in the picture. In this experiment, each participant repeated three trials, with a fourth trial being the baseline trial, throughout all of which the image of a hand without a ball was presented (60 s). We calculated the average of the data obtained from the first three trials, and then calculated the difference between that and the data of the fourth baseline trial with regard to the height of the hand. This was done because our pilot studies suggested that when participants repeated such trials, it might have become increasing easy to lower their hand as the trials progressed, even if sufficient rest was taken before each trial (as with the results of Experiment 1B), possibly because of muscle fatigue. We recorded the position of the tip of the middle finger in this experiment.

#### Results and discussion

**Figure 7:**
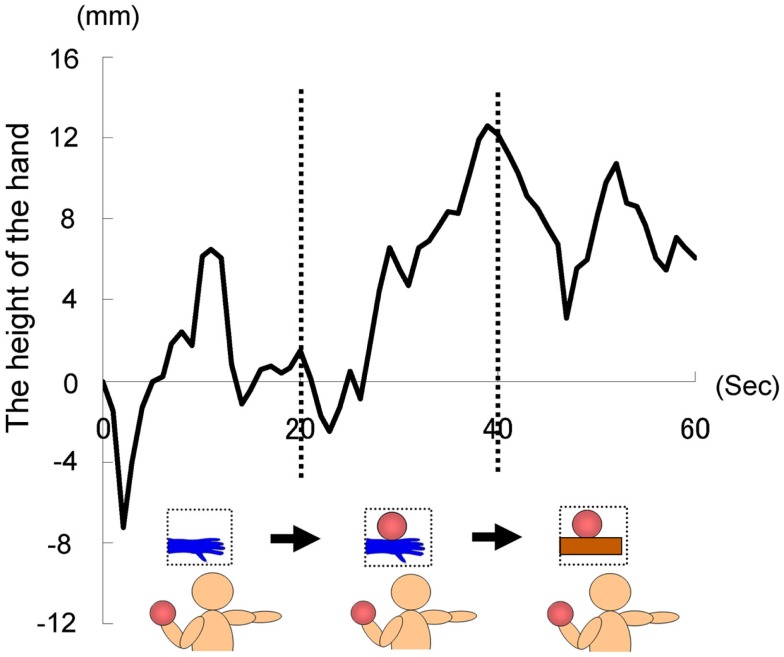
**Time course of the height of the hand in each condition in Experiment 2A**.

**Figure 8:**
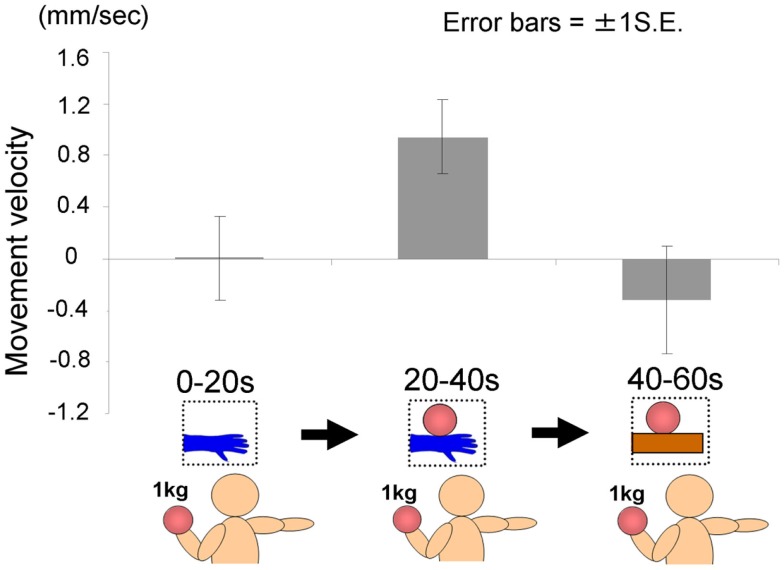
**Movement velocity in each condition in Experiment 2A**.

The time courses of the hand positions of the participants indicated that they could keep the hand almost immobile for the first 20 s (a model’s hand with no ball), then tended to raise the hand gradually for the next 20 s (a model’s hand with a ball), and then could again keep the hand almost immobile for the last 20 s (a ball on a wooden block; Figure 7). A one-way ANOVA (three visual stimuli conditions) was conducted to examine the movement velocity of the hand (Figure 8). These analyses demonstrated a significant main effect of condition [*F*(2.30) = 4.42, *p* < 0.05], and *post hoc* Ryan’s multi-comparison revealed significant differences between the first and second stimuli, and between the second and third stimuli (*p* < 0.05). These results suggest that the participants tended to raise the hand only while observing a weighted ball on a model’s hand, and not while observing a ball on a wooden block.

As hypothesized, the heaviness contagion was induced by observing a person, indicating that direct matching might be the underlying mechanism (Iacoboni et al., 1999) and that MNS is the underlying neural mechanism (Rizzolatti and Craighero, 2004). A hand-shaped object was not used because previous studies have shown that its reality (i.e., its similarity to a real person’s hand) might affect the simulation process of the observers (see General Discussion for detail). Although the present experiment suggested that an object shaped unlike a hand would not drive a feeling of heaviness in the observers, further research should address this issue (e.g., by using a wooden hand, a robotic hand, a xenogeneic hand, etc.). Although the current experiment suggests that the heaviness contagion as well as other motor simulation have a biological basis (Liepelt and Brass, 2010; Liepelt et al., 2010), previous studies, especially those in social psychology, have suggested that different people affect our simulation mechanisms differently (Calvo-Merino et al., 2006; Hein and Singer, 2008; Xu et al., 2009). The following final experiment examined the type of person, amongst a variety of people, who drives the heaviness contagion of observers.

## Experiment 2B

Finally, this experiment showed that a person who is similar to an observer could drive a feeling of heaviness in that observer; as in “like will to like.” We manipulated the visual appearance between a model’s hand and each participant’s hand. It was hypothesized that only those participants whose hand was similar to a model’s hand would be subject to heaviness contagion.

### Material and methods

#### Participants

A total of 24 participants (four males and 20 females, mean age = 19.5 years, range = 18–24 years) were randomly divided into two groups, both of whom saw a model’s hand holding a ball, and also held a visually identical ball in their left hands. Participants in the first group wore a blue-glove on their right hand, which was the same as the one that was worn on the model’s hand (this was called the blue-glove group), whereas those in the second group wore a yellow-glove (this was designated the yellow-glove group). Both gloves weighed 50 g.

#### Apparatus

The experimental device and environment were identical to those in Experiment 2A.

#### Procedure

As in previous experiments, we instructed each participant to hold the right hand, on which a glove was worn, in a horizontal position with their fingers stretched throughout the trial, which lasted 30 s. For the first 15 s, the image of a hand without the ball was presented. After 15 s, the image was changed to one of a hand holding a ball. All participants held a weighted ball (1 kg) in their left hand during each trial, so that they were aware of the weight of the ball in the picture. Each participant repeated three trials, with the fourth trial being the baseline trial, throughout all of which the image of a hand without a ball was presented (30 s), as in Experiment 2A. We recorded the position of the tip of the middle finger.

#### Results and discussion

**Figure 9:**
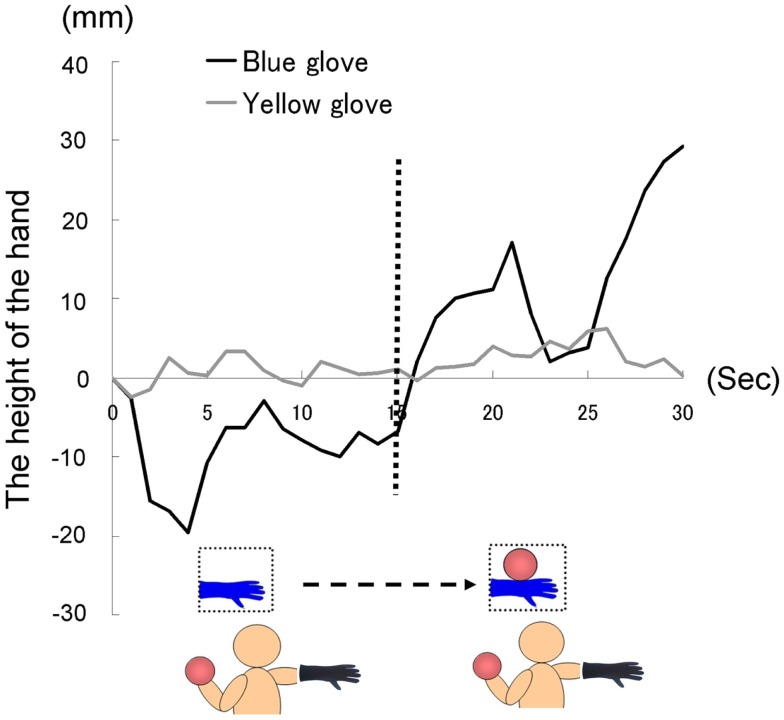
**Time course of the height of the hand in each group in Experiment 2B**.

The time courses of the hand positions of the participants indicated that those in both groups were capable of keeping the hand almost immobile for the first 15 s (a model’s hand with no ball); however, during the last 15 s (a model’s hand holding a ball), participants in the blue-glove group, who were wearing the same glove as worn on a model’s hand, tended to raise their hands, whereas those in the yellow-glove group kept the hand still and almost immobile (Figure 9).

**Figure 10:**
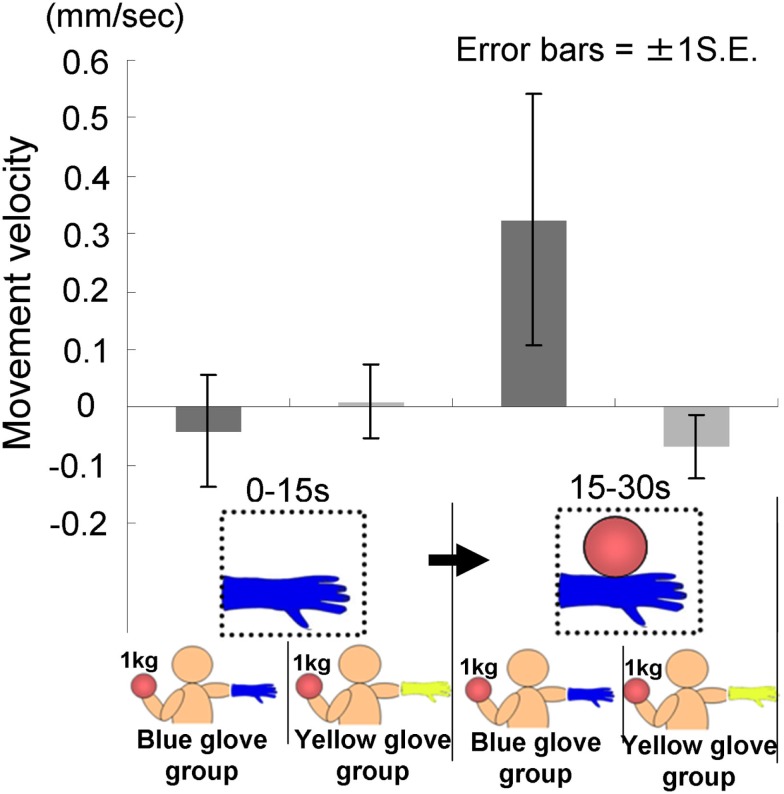
**Movement velocity in each group in Experiment 2B**.

We conducted a two-way ANOVA (two visual stimuli × two groups) to examine the movement velocity of the hand (Figure 10). These analyses demonstrated a significant interaction [*F*(1.22) = 5.53, *p* < 0.05], but no significant main effect of group [*F*(1.22) = 1.29, *p* > 0.20] or visual stimuli [*F*(1.22) = 2.30, *p* > 0.10]. The simple main effect of the group under the last visual stimuli (a model’s hand holding a ball) condition and the simple main effect of visual stimuli under the blue-glove condition were significant (*p* < 0.05). These results suggested that only participants who wore the same glove as that worn by the model tended to raise their hand while observing a model’s hand holding a weighted ball.

This suggested that we have specific targets for motor simulation, that is, a person who is “like me,” as suggested in some previous studies (see General Discussion for detail). In the present experiment, participants who wore a glove that was different from that worn by the model did not feel illusory heaviness on their hand, whereas in the previous experiments, although the participants did not wear a glove, they felt an illusory weight. This may seem contradictory in the sense that the hands of both sets of participants were visually different from the model’s hand. One reason for this may be that in the previous experiments, the participants perceived a model’s hand as a neutral hand wearing a glove (the hand was merely one of others), whereas in the present experiment, a model wearing a glove that is different from that worn by the participants may appear as a person explicitly defined as different from the participants themselves (the hand was one of others that differ from mine), thereby indicating in-group vs. out-group identification bias (see General Discussion). We shall now explain our findings using the mechanism behind motor simulation and how this may be construed as an expression of empathy.

## General Discussion

The results of the present study suggest that we may ourselves feel the heaviness felt by others, by observation alone (“heaviness contagion”). This new phenomenon might be driven predictively (i.e., in the present study, the participants predicted the feeling of heaviness experienced by another and raised their own hands), mandatorily (since they did not ignore it, participants in the present study needed to compensate for illusory heaviness; Experiment 1AB), and as a potential expression of empathy (the participants may have only responded to human counterparts, especially a person who was like them; Experiment 2AB). We shall discuss each factor with regard to extending motor simulation theory and the potential neural mechanism below.

### Simulation of others’ sensations is predictive

In our daily life, we can share many kinds of feelings with others, which may promote our social interaction as a social animal (see Iacoboni, 2009; Thioux and Keysers, 2010). Some previous studies have suggested that this ability has been learned through our previous experiences, which are underpinned by neural-based learning, such as experience-based Hebbian learning, or an internal model that forms links between the sensory processing of actions and motor plans (Iacoboni, 2009). Therefore, we appear to be able to simulate the action of others only when that action is also part of our own repertoires, especially with regard to skilled actions (Calvo-Merino et al., 2005; Lahav et al., 2007; Aglioti et al., 2008). Furthermore, we may also simulate the action or mental states of others, through prediction or generalization based on a learned model, if this action or mental state is not one that is particularly complicated, even if this is something not previously experienced. Patients with the rare syndrome of congenital insensitivity to pain showed normal fMRI responses to observed pain in the anterior mid-cingulate cortex and anterior insula (so-called shared circuits for pain experienced by both the self and others (Danziger et al., 2009), indicating that although they could not feel pain subjectively, they could predict the sensation of it, despite no previous experience of pain.

In general, how we feel depends on our predictions. This is true even if the target is not included in our repertoire, as long as it is simple. Size-weight illusion means smaller-sized objects feel heavier than larger-sized objects of the same weight, suggesting that we might predict weight from size, even for unfamiliar objects (Ross, 1966; Flanagan and Beltzner, 2000). In addition, we might see, hear, feel, taste, move, and perform as we predict (e.g., Barber and Calverley, 1964; Santarcangelo et al., 2005; Durgin et al., 2007; Plassmann et al., 2008; Castle et al., 2012). The present study suggested that this is also true in simulating others’ sensations; we might be resonant with others as we predicted (Iacoboni et al., 2005), indicating that motor simulation, which might be realized by action-perception coupling (James, 1890), is one of our basic processes, as with other perceptual functions. However, it only targets people (human agents), not objects (non-human agents). The reason for why this function could be driven through prediction is explained in the following discussion in terms of the target that we resonate with.

### Simulation of others’ sensations is mandatory

As a social animal, are we innately motivated to share feelings with others? Some previous studies have differentiated the brain activity that occurs between automatic and intentional empathy or imitation, by comparing only seeing (evaluating skin color) and actively sharing the feelings regarding the facial expressions of others (de Greck et al., 2012), or by comparing finger movements between only responding to a spatial cue and imitating that cue (Bien et al., 2009). Although these studies have suggested that we have an automatic and implicit function for simulation, “automatic” does not always mean “mandatory,” in the sense that we have a veto. It is possible that we could role-play the behaviors of others implicitly and automatically to promote our social communications. Some other studies reported that observing an action made by a human interferes with executed actions (Kilner et al., 2003, 2007). Although these studies have suggested that we do not ignore the observed actions of others, the possibility of demand characteristics of study participants, that is, the ability to speculate on the intention of the experimenters and to behave as expected remains, and therefore should be carefully controlled for, especially in this topic, because empathy or motor simulation could be linked with the estimation of the intention of others (i.e., mind-reading; Singer, 2006). Study participants may be resonant not with the stimuli, but with the experimenter (“experimenter effects”). A compensatory reaction to sensation transmitted from others is suggested by the results of the present study, and might mean that the participants did not ignore the sensation, regardless of the expectation of the experimenters, since they were doubly blind to the purpose (our expectation was neither that the hand could be kept immobile, nor that the hand might be lowered in response to heaviness felt).

This mandatory process might be underpinned by its potential neural mechanism. Because the MNS does not distinguish between external (others) and internal (self) action representation, it allows the individual to gain an experiential knowledge of the observed action in the absence of any motor output, as if that person is the agent of the action (Rizzolatti and Craighero, 2004). This indicates that we also need the process of distinguishing between representation of the action of the self and of others, such as the “who system” or the sense of agency or body ownership (Jeannerod and Pacherie, 2004; Schutz-Bosbach and Prinz, 2007) in order to inhibit such a mandatory contagion in situations such as those used in the present experiments. These functions might share the same circuit in our brain (Miall, 2003). This distinguishing mechanism could contribute to the compensatory reaction to feelings of heaviness. We can see that the participants totally disagreed, at least subjectively, with the assertion that a model’s hand on the screen appeared to be like their own hand (see Figure 4). They did not prevent the contagion from others, but simultaneously knew that it was not their own hand, which might lead to the need to adjust to the perceived illusory weight. This is not conclusive at the moment; however, it is essential to discuss self-other representation comprehensively in further research: simultaneously connecting and distinguishing between the functions of the self and others.

### Who is the target of our simulation?

Just as we do not constantly simulate, we also do not simulate everybody. Previous studies have suggested that the amplitude of empathic brain responses is modulated by the similarities between the self and others, such as gender, race, or previous experience, through observation (Calvo-Merino et al., 2006; Hein and Singer, 2008; Xu et al., 2009). A computational model-based approach explains that this is not only because of this sense of familiarity but also because individuals can predict the mental state or action representation of others, based on their own knowledge or learned model (Wolpert et al., 2003; Schutz-Bosbach and Prinz, 2007). Mirroring others might help to understand what another person is doing or feeling, or to predict what that individual is most probably going to do next (Blakemore and Frith, 2005; Iacoboni et al., 2005). Thus, this prediction is modulated by top-down processing, similar to animacy perception (Liepelt and Brass, 2010; Liepelt et al., 2010), the impossibility of the action (Longo et al., 2008), or spatial compatibility (Bertenthal et al., 2006). The similarities between observers and targets, even if it is a simple visual appearance as examined in the present study, might enhance an observer’s predictability of others for a simulation. The similarity effect may affect simulation responses through the tendency of an observer to identify more closely with others who appear to be similar to themselves, with regard to features such as personality, visual appearance, cultural likeness, sentience, or circumstance (Gruen and Mendelsohn, 1986; Brown et al., 2006), that is, in-group empathy (Rae Westbury and Neumann, 2008).

This may also be true of the difference between humans and other animals, or objects. It has been well documented that the MNS might be activated when observing conspecific counterparts (Gallese and Goldman, 1998), and, in line with this, some studies have suggested that the amplitude of empathic responses is also modulated by the phylogenetic similarity between the observers and their targets (Hills, 1995; Rae Westbury and Neumann, 2008). In addition, motor simulation has a biological bias (Press et al., 2005; Tsai and Brass, 2007; Watanabe, 2008; Liepelt and Brass, 2010; Liepelt et al., 2010), indicating that we do not simulate non-human agents. Nevertheless, other previous studies show that it is possible to be resonant with those who are different from us, such as people with different cultural backgrounds, animals, cartoon characters, and artificial objects, even early in life (Abell et al., 2000; Buccino et al., 2004; Hamlin et al., 2007; Perry et al., 2010). We can feel pain on the virtual or artificial hand (Ehrsson et al., 2007; Hägni et al., 2008), whereas observing an action made by a robot might not interfere with executed actions (Kilner et al., 2003). However, action-speed contagion might be driven by point-light biological motions (Watanabe, 2008) or the motor priming effect, which is an expression of motor simulation that is possibly modulated by beliefs about animacy or even virtualness of the hand (Longo and Bertenthal, 2009; Liepelt and Brass, 2010). Although it is also possible that biological tuning of motor simulation is highly action-selective (Liepelt et al., 2010), it might be presently difficult to form clear criteria for differentiating between the agents that we can be resonant with and the ones that we cannot. Nevertheless, since illusory body ownership of an artificial object might depend on its corporeality (Tsakiris et al., 2010), as the present study also suggested, we might again assume the importance of a similarity between observers and targets, which could make us feel closer to others (even animals or objects), and therefore to which we could apply our own knowledge. However, there is still a large gap between the lower level of self-other representation such as sensorimotor direct matching or motor simulation and the higher level of it such as top-down biological bias or in-/out-group empathy. Therefore, future research should tackle this problem in terms of social cognition (Farmer et al., 2012).

### Limitation of the current study

The present study suggested the new behavioral phenomenon of motor simulation in order to develop a background theory. The behavioral evidence of motor simulation, however, is not always compatible with neuroscientific or subjective report studies. Observing others’ action evokes the cortical activation (Iacoboni et al., 1999) but it does not evoke the execution of the movement; an exception is people with pathological conditions (see for review, Bertenthal et al., 2006). We can observe this through the facilitation in reaction time when observers do the same (e.g., Liepelt and Brass, 2010; Liepelt et al., 2010) or even unrelated action (Brass et al., 2000; Watanabe, 2008). Furthermore, our brain is activated in response to observed tactile stimuli to others (Keysers et al., 2004); however, except for specific people with mirror-touch synesthesia (Blakemore et al., 2005) who could have enhanced subjective empathy traits, we do not generally feel this tactility in reality (Banissy and Ward, 2007). As discussed, this may be because of the inhibition process that we possess to block automatic contagion. Therefore, to increase the behavioral response of study participants, our experimental methodology used a unique procedure: a ball was held during trials, and not just felt its heaviness before trials. This might give a potential artifact, although this was carefully controlled for in our experiments (that is, a potential effect of holding a ball: see Experiment 1A). Further studies should refine what information would be needed from others, as well as how and when it is needed, in order to elicit heaviness contagion.

## Conflict of Interest Statement

The authors declare that the research was conducted in the absence of any commercial or financial relationships that could be construed as a potential conflict of interest.
